# IRE1α and IGF signaling predict resistance to an endoplasmic reticulum stress-inducing drug in glioblastoma cells

**DOI:** 10.1038/s41598-020-65320-6

**Published:** 2020-05-20

**Authors:** Jeffrey J. Rodvold, Su Xian, Julia Nussbacher, Brian Tsui, T. Cameron Waller, Stephen C. Searles, Alyssa Lew, Pengfei Jiang, Ivan Babic, Natsuko Nomura, Jonathan H. Lin, Santosh Kesari, Hannah Carter, Maurizio Zanetti

**Affiliations:** 1 The Laboratory of Immunology, Department of Medicine and Moores Cancer Center, University of California, San Diego 9500 Gilman Drive, La Jolla, CA 92093-0815 USA; 2 Division of Medical Genetics, Department of Medicine, University of California, San Diego, 9500 Gilman Drive, La Jolla, CA 92093 USA; 3 Department of Cellular and Molecular Medicine, University of California, San Diego, La Jolla, California USA; 4 Department of Translational Neurosciences and Neurotherapeutics, John Wayne Cancer Institute/Pacific Neuroscience Institute, 2200 Santa Monica Boulevard, Santa Monica, CA 90404 USA; 5 Department of Pathology, Stanford University, Palo Alto, CA 94305 USA

**Keywords:** CNS cancer, CNS cancer

## Abstract

To date current therapies of glioblastoma multiforme (GBM) are largely ineffective. The induction of apoptosis by an unresolvable unfolded protein response (UPR) represents a potential new therapeutic strategy. Here we tested 12ADT, a sarcoendoplasmic reticulum Ca^2+^ ATPase (SERCA) inhibitor, on a panel of unselected patient-derived neurosphere-forming cells and found that GBM cells can be distinguished into “responder” and “non-responder”. By RNASeq analysis we found that the non-responder phenotype is significantly linked with the expression of UPR genes, and in particular *ERN1* (IRE1) and *ATF4*. We also identified two additional genes selectively overexpressed among non-responders, *IGFBP3* and *IGFBP5*. CRISPR-mediated deletion of the *ERN1, IGFBP3, IGFBP5* signature genes in the U251 human GBM cell line increased responsiveness to 12ADT. Remarkably, >65% of GBM cases in The Cancer Genome Atlas express the non-responder (*ERN1, IGFBP3, IGFBP5*) gene signature. Thus, elevated levels of IRE1α and IGFBPs predict a poor response to drugs inducing unresolvable UPR and possibly other forms of chemotherapy helping in a better stratification GBM patients.

## Introduction

Glioblastoma (GBM) is a devastating, rapidly fatal disease whose survival rate has not improved much in recent years relative to other tissues. With the current standard of care for newly diagnosed GBM of surgical resection followed by temozolomide and radiotherapy, the expected median survival remains under two years^1^. This inadequacy leaves open the necessity for novel therapeutic approaches targeting the signaling programs GBM cells rely on to acquire chemoresistance and survive in the face of various challenges in the tumor microenvironment, e.g., hypoxia, radiation therapy, and chemotherapy (temozolomide).

In mammalian cells the unfolded protein response (UPR) represents a powerful homeostatic signaling mechanism and an adaptive cellular response to the accumulation of mis- or unfolded protein within the endoplasmic reticulum (ER)^2^. This homeostatic mechanism regulates the balance between cell survival and apoptosis such that if adaptation/restoration to proteostasis fails, the apoptotic program is activated^2^. This evolutionarily conserved signaling complex is mediated by three initiator/sensor ER transmembrane molecules: inositol-requiring enzyme 1 (IRE1α), PKR-like ER kinase (PERK), and activating transcription factor 6 (ATF6), which, in the unstressed state, are maintained in an inactive state through association with 78 kDa glucose-regulated protein (GRP78)^3^. Upon activation of the UPR, PERK phosphorylates eIF2α, resulting in the selective inhibition of translation. Contextually, IRE1α autophosphorylates to activate its endonuclease domain, resulting in the cleavage of *Xbp-1* to generate a spliced Xbp-1 isoform (Xbp-1s), which drives the production of various ER chaperones to restore ER homeostasis. IRE1α‘s RNase domain can also cause endonucleolytic decay of many ER-localized mRNAs through a phenomenon termed regulated IRE1-dependent decay (RIDD)^4^. ATF6 translocates to the Golgi where it is cleaved into its functional form, and activates transcriptionally XBP1 to restore ER homeostasis^5^. In solid tumors the UPR develops in response to special local environmental conditions such as nutrient deprivation, hypoxia, oxidative stress, but also viral infection (e.g., HBV, HCV, and HPV) or genomic abnormalities such as aneuploidy^6^.

Unlike untransformed somatic cells, tumor cells are already programmed for self-renewal and resistance to DNA damage through the activation of telomerase^7,8^. Consequently, using cell-autonomous or cell-nonautonomous mechanisms, tumor cells leverage the UPR to further adapt to unfavorable microenvironmental conditions and develop resistance to therapy^9–11^. GBM tumor aggressiveness and chemoresistance correlates with elevated levels of GRP78^12^ or IRE1α^13,14^, but not PERK. Furthermore, XBP1 splicing or RIDD activation have been found to correlate with different GBM phenotypes and tumor growth characteristics, suggesting that single UPR elements are points of vulnerability that could be exploited therapeutically to cause cell death and tumor arrest^15^. However, since no FDA-approved drugs exist to inhibit a specific UPR pathway^16^, an attractive alternative therapeutic approach is to induce substantial ER stress to drive the UPR’s apoptotic, rather than adaptive, signaling^17^. This can be realized, for instance, through the inhibition of the sarcoendoplasmic reticulum calcium transport ATPase (SERCA), which ensues in an acute depletion of Ca^++^ in the ER and the induction of a supra-physiological UPR. The pro-drug G-202 can accomplish this by releasing the active component 12ADT, a thapsigargin analogue, upon activation^18^.

Based on this reasoning, we studied the responsiveness of patient-derived GBM neurospheres to 12ADT. Strikingly, we found unique transcriptional signatures distinguishing responder from non-responder phenotypes. We further investigated the genes contained within these signatures for their relative contribution to 12ADT mediated cytotoxicity. These results provide novel insights into the transcriptional networks of GBM cells in relation to their sensitivity to treatment, hence establishing new predictive criteria for the treatment of patients with GBM.

## Results

### GBM cells respond differentially to 12ADT

**Figure 1:**
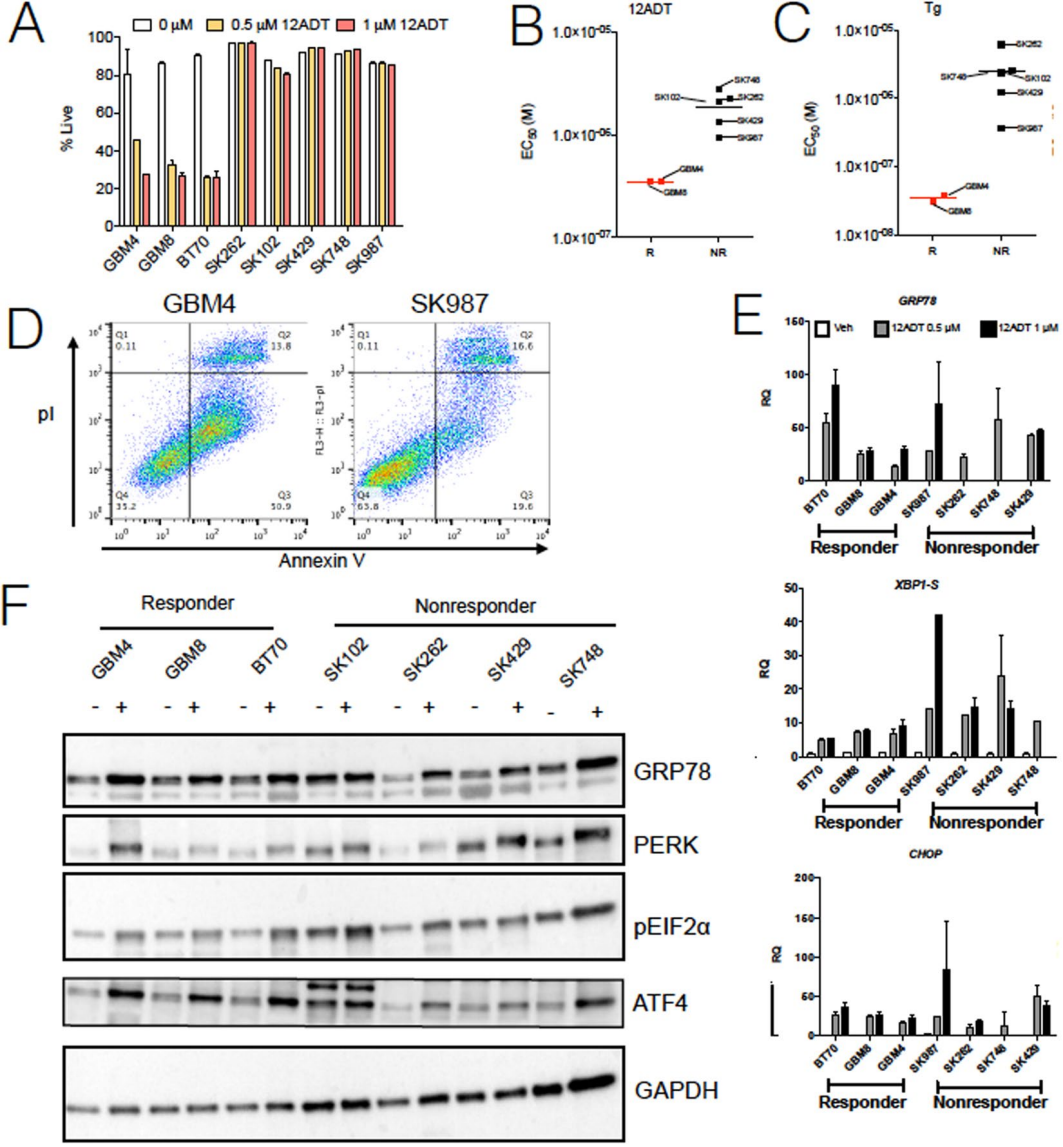
Differential toxicity of 12ADT across neurosphere lines. (**A**) Flow cytometric determination of GBM neurosphere line survival as determined by 7AAD positivity after 48 hour treatment with specified concentration of 12ADT. *P ≤ 0.05, ***P ≤ 0.001, Student’s t test (paired two-tailed). These results are representative of two independent experiments. Distribution of EC_50_ values between Responder (R) and Nonresponder (NR) neurosphere lines treated with either (**B**) 12ADT or (**C**) thapsigargin (Tg) for 72 hours and processed for viability by Alamar Blue absorbance. (**D**) Annexin V apoptosis staining of representative responder (GBM4) and nonresponder (SK987) after 48 hour treatment of 12ADT at 1 μM. (**E**) RT-qPCR of UPR associated genes in GBM neurosphere lines with specified concentration of 12ADT after 48 hour treatment (n = 2). Gene expression was normalized to each line’s respective 0 µM condition to determine relative quantification (RQ). (**F**) Western blot analysis of GBM neurosphere cell lines treated (+) or untreated (−) with 12ADT at 0.5 μM after 48 hours. Each data point is a single experiment with three replicates, and is representative of two independent experiments.

A new and potentially effective approach to drive glioblastoma (GBM) cells to apoptosis is to induce an acute and unresolvable ER stress response. Mipsagargin (G-202) is a prodrug that is hydrolyzed by prostate specific membrane antigen (PSMA), which is highly expressed in the stroma of 75% of brain tumors^19^ relative to normal brain tissue. PSMA hydrolysis releases G-202’s active component, 8-O-(12-aminododecanoyl)-8-0 debutanoylthapsigargin (12ADT), a synthetic analogue of thapsigargin, which through its inhibition of the sarcoendoplasmic reticulum Ca^2+^ ATPase (SERCA) is a canonical and potent inducer of ER stress^20^. A potent *in situ* dose of 12ADT generates unresolvable ER stress in tumor cells driving a pro-apoptotic UPR. Thus, 12ADT could serve as novel chemotherapeutic to drive apoptosis in GBM cells. To test this hypothesis, we treated eight unique patient-derived GBM neurosphere forming cell lines for 48 hours with low doses (0.5–1 µM) of 12ADT and probed survival through flow cytometric detection for incorporation of the cell death marker, 7-aminoactinomycin D (7AAD) (Fig. 1A).

We found a striking variation in sensitivity to 12ADT across the eight neurosphere lines, with three being very sensitive (GBM4, GBM8, BT70) and five being resistant (SK102, SK262, SK429, SK748, SK987). Hereunder, we refer to these two groups as responder (R) and non-responder (NR). To investigate these results further, we treated R and NR neurosphere lines with increasing concentrations of 12ADT over 72 hours and determined EC_50_ values based on the incorporation of Alamar Blue stain for cell viability (Fig. 1B). The use of thapsigargin instead of 12ADT produced comparable results, suggesting that the R and NR phenotypes are due to SERCA inhibition (Fig. 1C). Importantly, the prodrug itself (G-202) did not induce substantial cytotoxicity in a R neurosphere line, likely due to their low PSMA expression (Supplementary Fig. 1). Also, we could not attribute the different response to 12ADT to mutations in the SERCA gene. We used a Genome Analysis Toolkit (GATK)^21^ to call variants in RNASeq data and did not find biases in the SERCA gene except one mutation in SK429 cells, suggesting that the R and NR phenotypes are not function of variability in the SERCA gene. Annexin V staining in representative R and NR neurosphere lines confirmed that 12ADT induced apoptosis in a large proportion of R cells (Fig. 1D), consistent with the known mechanism of UPR mediated cell death^2^.

On the basis of the difference in 12ADT-induced cytotoxicity between R and NR neurosphere lines, we hypothesized the involvement of different UPR genes in these groups. We found no significant differential expression in the key UPR genes *GRP78, CHOP*, and spliced *XBP-1* (*XBP-1s*) between R and NR neurosphere lines after a 48 hour treatment with 12ADT (Fig. 1E). Next, we interrogated the expression of several UPR proteins by Western blot. We began with the master regulator of the UPR, *GRP78* (*HSPA5*), the expression of which correlates with poorer survival of GBM patients (Supplementary Fig. 2) and was previously reported to confer chemoresistance in glioma cells^13^. We also analyzed the PERK pathway due to its role in chemoresistance in prostate cancer cells^11^ and its central role in coordinating UPR-associated apoptosis. While expression of these targets increased upon 12ADT treatment, we again did not find an expression pattern distinguishing R and NR neurosphere lines (Fig. 1F). Collectively, these data suggest that the differential susceptibility to 12 ADT treatment could not be explained by the perturbation of the expression of a single UPR element alone.

### Differential UPR signatures in responder and non-responder neurospheres

**Figure 2:**
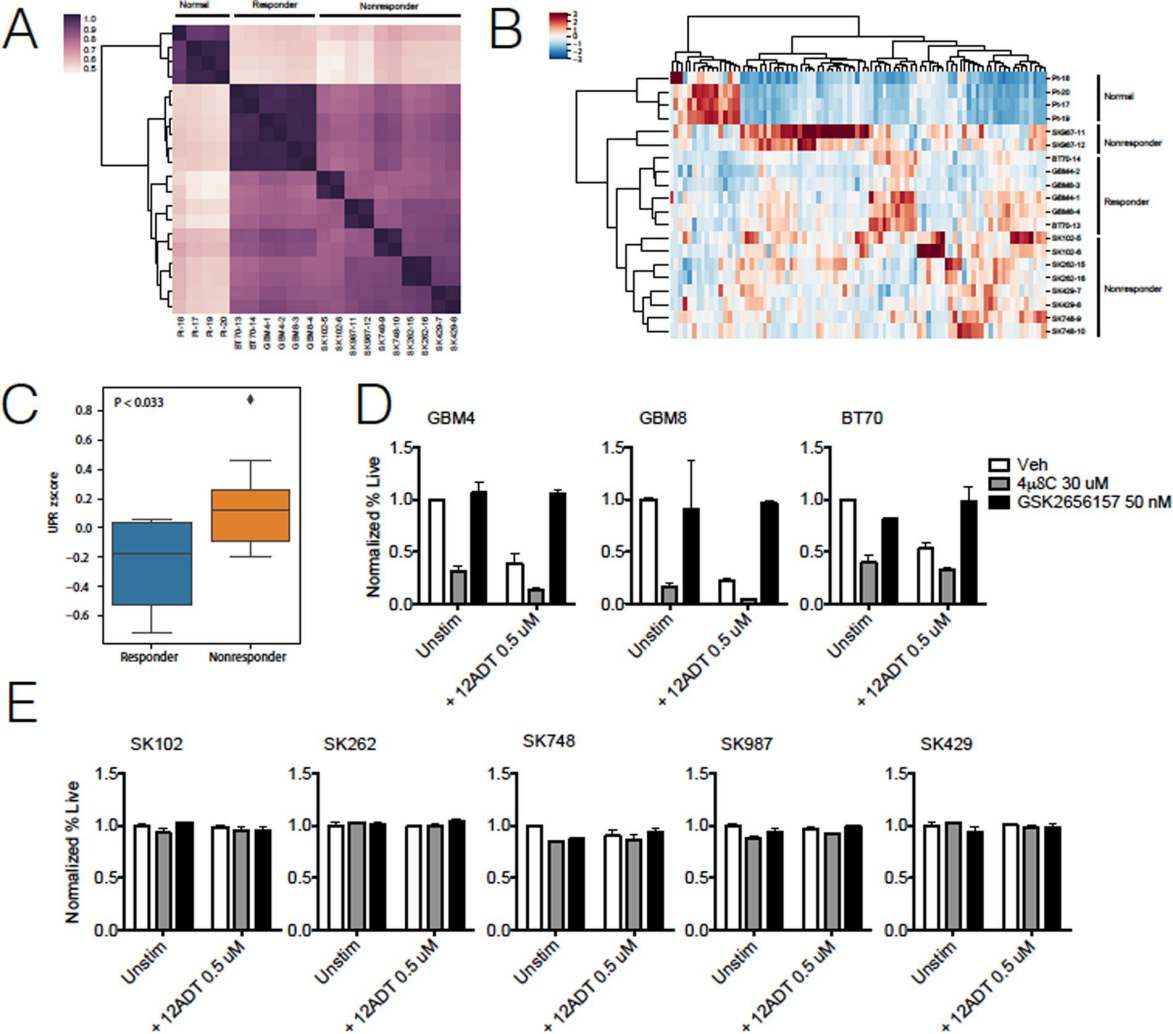
Differential expression of UPR genes predicts 12ADT sensitivity. (**A**) Whole transcriptome-based unsupervised clustering of neurosphere lines and normal control samples. Normal and responder samples form distinct clusters, while nonresponder neurosphere lines cluster more weakly, suggesting more inter-sample transcriptional heterogeneity. (**B**) A heatmap displaying sample clustering based on the z-scored expression of 85 UPR genes. Unsupervised hierarchical clustering based on UPR gene expression grouped samples according to status with a single outlier among nonresponder neurosphere lines (SK987). (**C**) Comparison of aggregated z-scored UPR gene expression shows that the UPR is overall elevated in nonresponder neurosphere lines relative to responder lines. Normalized cell survival of (**D**) responder or (**E**) nonresponder neurosphere lines treated with 12ADT (0.5 μM) in the absence or presence of IRE1α inhibition (4μ8C) or PERK inhibition (GSK2656157). Survival determined by flow cytometry for 7AAD negativity and normalized by percent live (7AAD^−^) population of unstimulated, inhibited condition (n = 2 per group). *P ≤ 0.05, **P ≤ 0.01, Student’s t test (paired two-tailed). Data representative of three independent experiments.

The absence of clear differences in GRP78 and PERK-associated proteins in R versus NR neurosphere lines led us to consider a more complex expression signature distinguishes the R/NR phenotypes. To that end, we isolated total RNA from the eight GBM neurosphere lines grown under homeostatic cell culture conditions (i.e., without treatment) as well as healthy brain tissue to serve as a negative control and performed RNASeq analysis. First, we evaluated the transcriptional similarity across replicates. We found that normal healthy controls clustered separately from the neurosphere lines, and that R neurosphere lines clustered more tightly than NR neurosphere lines (Fig. 2A). In a transcriptome-wide analysis, we also found distinct clusters by principal component analysis (Supplementary Fig. 3). We next interrogated the expression of 85 UPR-associated genes (Supplementary Table 1) across samples and found that an unbiased hierarchical clustering was able to distinguish R neurosphere lines unambiguously from NR (Fig. 2B). When we examined UPR activity at the global level on the basis of an aggregate z-score of all eighty-five genes in this pathway, we found that the cumulative expression of UPR genes was higher in NR compared to R neurospheres lines (p < 0.033; Fig. 2C). From this global analysis, we sought to identify individual genes that were driving this observation. We found that NR neurosphere lines had a significantly higher expression in 19 UPR associated genes after multiple testing correction, including *ATF4* (p < 0.04) and *ERN1* (p < 0.05) whereas nine other genes were overexpressed in R neurosphere lines relative to NR (Supplementary Table 1). Transcripts for genes in the ATF6 pathway, including *ATF6* (p > 0.93) itself and its upstream activator *GRP78* (p > 0.33), showed no significant differences between R and NR neurosphere lines, suggesting that this pathway played no central role in 12ADT sensitivity. We also found no significant difference in *XBP1* (p > 0.66) expression. We conclude that under homeostatic conditions, R and NR neurosphere lines possess unique genetic UPR signatures, and that the increased constitutive expression of two genes - *ATF4* and *ERN1*- is associated with increased resistance to 12ADT cytotoxicity.

We were intrigued that both *ERN1* (the gene coding for IRE1) and *ATF4* were increased in the NR group over the R group. Recent reports have suggested that *ERN1* drives a variety of tumorigenic characteristics such as adhesion/migration and inflammation and correlates with reduced survival in GBM patients^15^. Another report suggested a functional link between expression of *ATF4* and *ERN1* activity^22^. Therefore, we posited that inhibiting the IRE1 pathway would increase sensitivity to 12ADT in NR neurosphere lines. To test this hypothesis, we evaluated the relative roles of IRE1α and PERK pathways in the sensitivity of neurosphere lines to 12ADT using pharmacological inhibition with 4μ8C and GSK2656157. 4μ8C^19^ is an IRE1α RNAse inhibitor, and GSK2656157^20^ inhibits PERK autophosphorylation and consequently attenuates the expression of the downstream genes *ATF4* and *CHOP*. We treated the R and NR neurosphere lines with each inhibitor in the absence or presence of 12ADT for 48 hours and measured cell viability by flow cytometry. GSK2656157 treatment protected R neurosphere lines from 12ADT-mediated cell death, perhaps owing to the established role of the PERK pathway in mediating ER induced apoptosis (Fig. 2D). On the other hand, we found that treatment with 4μ8C alone reduced the viability of all three R neurosphere lines and increased their sensitivity to 12ADT (Fig. 2D). Surprisingly, 4μ8C alone or in combination with 12ADT at moderate concentrations (0.5 μM) did not affect 12ADT sensitivity in NR neurosphere lines (Fig. 2E). Together, these results imply that IRE1α RNase activity promotes resistance to 12ADT in R, but not NR neurosphere lines.

### A link between the UPR and insulin growth factor (IGF-1) signaling

**Figure 3:**
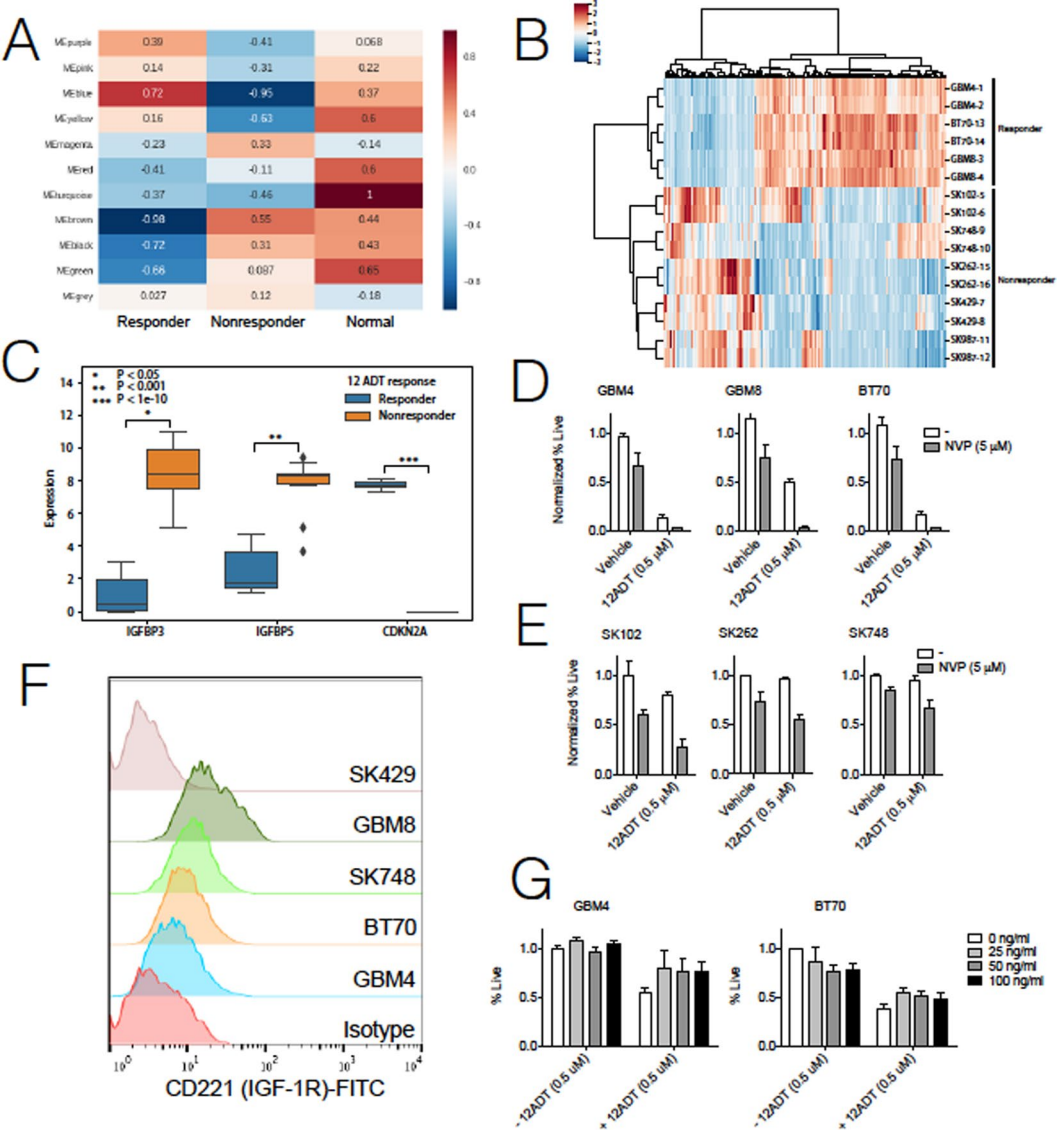
IGF signaling contributes to 12ADT toxicity. (**A**) Heatmap displaying Pearson correlation coefficients between eigengene profiles and sample traits for modules detected by WGCNA. Samples were divided into 2 groups according to responder or control status before evaluating correlation. MEBlue and MEBrown modules display the strongest opposing correlation between responder and nonresponder neurosphere lines. (**B**) Heatmap of gene expression across samples for genes belonging to the MEBlue module shows differential expression of genes relative to response. (**C**) Comparison of expression levels between responder and nonresponder neurosphere lines of selected genes that show strong differential expression in MEBlue (IGFBP3, IGFBP5) and MEBrown (CDKN2A). (**D**) Normalized cell survival of three responder (GBM4, GBM8, BT70) and (**E**) three nonresponder neurosphere (SK102, SK262, SK748) lines treated with 12ADT (0.5 μM) in the absence or presence of IGF-1R inhibition (NVP-AEW541). Survival determined by flow cytometry for 7AAD negativity and normalized by percent live (7AAD^−^) population of unstimulated, inhibited condition (n = 2 per group) and are representative of three independent experiments. *P ≤ 0.05, **P ≤ 0.01, Student’s t test (paired two-tailed) (**F**) Detection of surface expression of CD221 (IGF-1R) in GBM neurosphere lines as determined by flow cytometry. (**G**) Normalized cell survival of responder cell neurosphere lines treated with 12ADT (0.5 μM), supplemented with increasing concentrations of IGF1, after 48 hour treatment. Survival determined by flow cytometry for 7AAD negativity and normalized by percent live (7AAD^−^) population of unstimulated condition where no IGF-1 supplementation occurred (n = 2 per group).

Because neither inhibition of the IRE1α or PERK pathways accounted for a differential sensitivity in NR neurosphere lines, we looked beyond UPR associated genes and performed a weighted gene correlation network analysis (WGCNA) to identify putative gene signaling networks and assess their roles in 12ADT responsiveness^23^. This WGCNA analysis detected modules of co-expressed genes and assigned an eigengene score for each module to represent its gene expression profile^23^. We then assigned Pearson correlation coefficients for each module’s eigengene expression across the experimental groups, including R and NR GBM neurosphere lines and healthy brain tissue (Fig. 3A). This analysis identified two modules, MEBlue and MEBrown, which showed the strongest opposing correlation coefficients for R versus NR status. These modules comprised genes associated with epithelial cell motility (MEBlue) (Fig. 3B) and protein metabolic processes (MEBrown), respectively (Supplementary Fig. 4). Further investigation of the most differentially correlated genes within these modules identified cyclin dependent kinase inhibitor 2 A (*CDKN2A*) along with two genes in the insulin growth factor 1 (IGF-1) signaling pathway, IGF binding protein 3 (*IGFBP3*) and IGF binding protein 5 (*IGFBP5*) (Fig. 3C). *CDKN2A* (p < 1e-10) was expressed at much higher levels in R relative to NR neurosphere lines (Fig. 3C, Supplementary Fig. 5). However, since this gene is commonly deleted in GBM^24^, we chose to pursue targets that would be more translationally relevant, since *CDKN2A* is likely undruggable. Specifically, *IGFBP3* (p < 0.05) and *IGFBP5* (p < 0.001) showed increased expression in NR compared to R neurosphere lines (Fig. 3C). Therefore, we hypothesized that IGF-1-mediated signaling may be involved in resistance to 12ADT. To that end, we treated R and NR neurosphere lines with 12ADT in the absence or presence of the IGF-1 receptor (IGF-1R) inhibitor NVP-AEW541^25^ for forty-eight hours and determined cell viability. While NVP-AEW541 was relatively well tolerated in both R (Fig. 3D) and NR (Fig. 3E) groups, its addition enhanced 12ADT-mediated cytotoxicity both in R and NR neurosphere lines.

Although the exact relationship between IGF-1R and the UPR is not well established, GRP78 was recently identified as a downstream target of IGF-1R signaling^26^, suggesting that GRP78 could mediate anti-apoptotic and growth promoting effects. To test this possibility, we performed surface staining for IGF-1R (CD221) on R and NR neurosphere lines but found no clear pattern distinguishing the two groups (Fig. 3F), suggesting that surface expression of IGF-1R does not contribute to differential responsiveness to 12ADT during IGF-1R inhibition. To determine if bioavailability of IGF-1 affects 12ADT resistance, we treated R neurosphere lines with 12ADT in medium supplemented with increasing concentrations (25, 50, 100 ng/ml) of IGF-1. The addition of IGF-1 to R neurosphere lines increased resistance to 12ADT but only moderately (Fig. 3G).

### CRISPR validates the role of *ERN1* and *IGFBP3/5* in the NR phenotype

**Figure 4:**
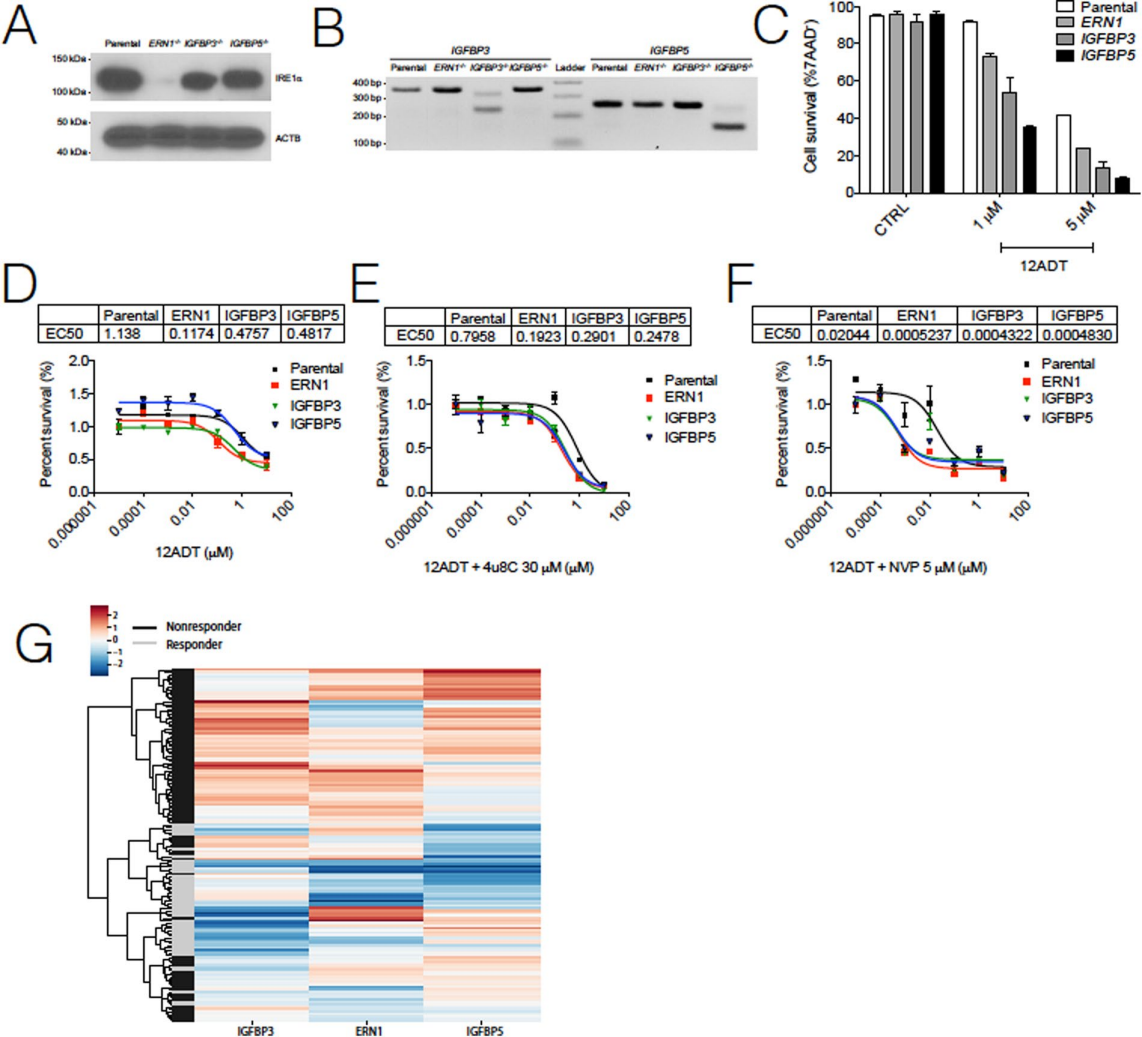
Loss of *ERN1, IGFBP3, IGFBP5* sensitize cells to 12ADT toxicity. (**A**) Western blot detection of IRE1 in U251 parental and CRISPR deleted *ERN1, IGFBP3, IGFBP5*. (**B**) PCR detection of *IGFBP3* and *IGFBP5* of U251 parental and CRISPR deleted *ERN1, IGFBP3, IGFBP5*. (**C**) Percent survival of U251 parental (WT) or CRISPR U251 cells during increasing concentrations of 12ADT after 48 hours. Survival determined by flow cytometry for 7AAD negativity (n = 2 per group) and are representative of at least three independent experiments. *P ≤ 0.05, **P ≤ 0.01, ***P ≤ 0.001 Student’s t test (paired two-tailed). (**D–F**) Normalized percent survival and corresponding EC50 values of U251 parental or CRISPR cell lines titration of 12ADT after 48 hours in absence (**D**) or presence of IRE1α inhibition (**E**) or IGF-1R inhibition (**F**) (n = 3 per condition). Percent survival determined by Alamar Blue absorbance values (n = 3) normalized at 12ADT 0.00001 μM absorbance value for each line. (**G**) Predicted 12ADT response status for 143 GBM samples in the TCGA GBM dataset.

Collectively, the RNA-Seq expression and pharmacological inhibition studies suggested that IRE1α and IGF-1 signaling contributed to 12ADT sensitivity. To further validate the contribution of these pathways to 12ADT-mediated cytotoxicity, we designed CRISPR guides against three target genes (*ERN1, IGFBP3, IGFBP5*) and transfected them separately into U251 glioblastoma cells. The change from patient derived neurosphere lines to immortalized human glioblastoma U251 line became necessary due to very poor transfection efficiency of the neurosphere lines. We confirmed gene knockout using Western blotting (Fig. 4A) and PCR on genomic DNA (Fig. 4B). The deletion of each of these genes augmented 12ADT cytotoxicity relative to the parental line across increasing concentrations of 12ADT, with the strongest effect observed in the *IGFBP5* knockout (Fig. 4C). To further substantiate these observations and also identify any cross-functionality among *ERN1, IGFBP3, IGFBP5*, we treated U251 CRISPR-deleted clones with 12ADT in the absence (Fig. 4D) or presence of 4μ8C (Fig. 4E) or NVP-AEW541 (Fig. 4F). Whereas 4μ8C treatment slightly increased the 12ADT EC_50_ in *ERN1−/−* clone (1.6-fold), it slightly decreased the EC_50_ in the parental U251 line (1.4-fold) and in both IGFBP clones (1.6-fold for *IGFBP3−/−*, and 1.9-fold for *IGFBP5−/−*). This is perhaps due to the effect of 4µ8c on other target mRNAs. Strikingly, the inhibition of IGF-1R with NVP-AEW541 dramatically enhanced 12ADT cytotoxicity for each knockout cell line (56-fold for parental, 224-fold for ERN1*−/−*, 1101-fold for *IGFBP3−/−*, and 997-fold for *IGFBP5−/−*). These observations demonstrate how important these two new pathways are in the response of GBM cells to UPR-inducing stressors.

### Estimating the generalizability of 12ADT response

Collectively, our results predict that GBM patients with lower *ERN1, IGFBP3*, and *IGFBP5* expression would respond more favorably to a UPR-based treatment such as G-202/12ADT. To determine what fraction of GBM patients exhibit such a gene expression profile, we probed one hundred and forty-three GBM samples within The Cancer Genome Atlas (TCGA). We trained a simple support vector machine (SVM) classifier on z-scored expression levels of *ERN1, IGFBP3*, and *IGFBP5* in R and NR neurosphere lines, treating replicates as independent samples. Given the small sample size (n = 16) available for training, the resulting classifier may provide a somewhat biased estimate on potential response. Using a cross validation approach where both replicates of each sample were withheld from the training, the classifier was able to classify responder status correctly in the remaining fourteen out of sixteen samples. We then applied this SVM classifier to GBM samples from TCGA (n = 143) (Fig. 4G). Our classifier suggested that 34% (n = 49) of GBM tumors could potentially be sensitive to UPR-based therapies, whereas the remaining 66% would require additional interventions targeting IGF-1 signaling or IRE1α signaling. Finally, we performed survival analysis using the progression free survival (PFS) data for GBM 94 samples from TCGA. Since lower expression was associated with response to treatment, we speculated that lower expression of these genes in general could indicate less aggressive disease. Of the three genes, the *IGFBP3* low group had a significantly better survival status than the *IGFBP3* high group (p = 0.03; one-tailed long-rank test; Supplementary Fig. 6A). Differences in progression free survival of the other two genes did not reach significance (Supplementary Fig. 6B).

## Discussion

Glioblastoma is one of the most aggressive solid malignancies whose survival rate has not improved much in recent years relative to other malignancies. Beside conventional approaches (surgery, radiation, and temozolomide), recent intervention attempts have leveraged immunotherapy. In this context, personalized neoantigen vaccination has met with immunological but not clinical responses^27,28^ and immune checkpoint inhibition^29^ is most applicable to patients with mismatch repair (MMR) deficiencies, which are present in a very low percentage of GBM patients^30^. Immunotherapy also faces additional hurdles such as local immune suppression by macrophage-like cells^31^ and immune evasion due to PD-L1-expressing exosomes released by GBM cells^32^. Thus, alternative approaches are needed.

A new conceptual therapeutic approach in GBM is the induction of apoptosis by exacerbation of the UPR’s apoptotic signaling through the creation of massive, unresolvable ER stress. Although 12ADT is not likely to cross the blood brain barrier, the prodrug Mipsagargin (G-202), which targets the tumor vasculature is in clinical trial (NCT01056029)^33^. Using an unselected panel of patient-derived GBM neurosphere lines, we found that susceptibility to apoptosis by a SERCA inhibitor analogue of thapsigargin clearly distinguishes two populations of responder (R) and non-responder (NR) conditions, which were also distinguished in the same manner by temozolomide treatment (Supplementary Fig. 7). Characteristic of the NR phenotype was a significantly higher expression of 19 UPR genes including *ERN1* and *ATF4* compared to the R phenotype. The tumor promoting role of *ERN1* is consistent with previous reports that IRE1α activity correlates in a cause-effect relationship with GBM aggressiveness^14,15,34^. Thus, heightened levels of IRE1α, a pro-survival factor^35^, causes resistance to UPR-induced apoptosis in GBM neurosphere lines.

Remarkably, transcriptional profiling and network analysis of R vs. NR neurosphere lines identified additional differentially-expressed gene networks. Within these, two genes, *IGFBP3* and *IGFBP5*, belonging to the IGF-1 signaling pathway, have been previously implicated in tumorigenesis^36,37^ including GBM^38^. IGFBPs can drive tumorigenesis by increasing IGF-dependent signaling that in turn increases cancer cell proliferation and survival^39^. Of interest, Ecuadorian people with the Laron syndrome, a rare form of short stature with a mutation in the growth hormone (GH) receptor and extremely low IGF-1 serum levels have very low susceptibility to cancer^40^, stressing a central role for the IGF-IGFBP axis in fueling cell proliferation and opposing apoptosis in cancer cells. Of note also is the fact that IGF-1 signaling positively regulates the expression of GRP78^26^, consistent with the fact that *HSPA5* (GRP78) expression in TCGA correlates with poorer survival of GBM patients (Supplementary Fig. 2). In addition, IGF-1 could potentiate replicative activity of GBM cells by stimulating telomerase^41^. Since *TERT* promoter mutations occur in 80% of GBM^42^, causing *per se* an increase in *TERT* transcription, the present data suggest that a constitutively high IGF-1 signaling could contribute to high telomerase activity in GBM cells.

We found that NR neurosphere lines had very low expression levels of *CDKN2A* compared to R neurosphere lines, implying a direct correlation between *CDKN2A* status and responder potential. However, separate inhibition of either IRE1α or IGF-1 signaling increased sensitivity to 12ADT, suggesting an unanticipated interdependence between the IRE1α pathway and the IGF-1 axis. Therefore, a homozygous deletion of *CDKN2A* in chromosome band 9p2, which is present in ~58% of GBM cases^24^, should have little role in attempts to convert a NR phenotype to a R phenotype.

In conclusion, the analysis of patient-derived GBM neurosphere lines enabled us to accurately profile responder vs. non-responder phenotypes. We found that sensitivity to a UPR inducing prodrug may be hindered not only by constitutively-high levels of activation of the UPR but also by a heightened expression of genes encoding proteins central to IGF-1 signaling. Remarkably, the NR signature was validated in TCGA and found to account for>65% of GBM cases. Therefore, new UPR-based therapies need to take into consideration the role of elevated IRE1α and IGFBPs making it urgent to develop strategies to disable them, thereby decreasing resistance and increasing sensitivity to UPR therapies. It may be important to verify if the NR signature identified herein can also be used to predict other forms of treatment.

## Materials and Methods

### GBM tissue acquisition, processing, and culture of GSCs

We have previously published on the origin and methods to generate the neurosphere lines used in this paper^44^. Briefly, GBM (grade IV glioma) tumor samples were obtained from adult human (>21 years) surgical patients without the exclusion of either sex or any ethnic/racial groups, under an approved UC San Diego Moores Cancer Center IRB (#IRB # 100936) protocol, with written, informed patient consent. These patient derived cell lines were made in Dr. Santosh Kesari laboratory under the protocol # identified herein. IRB ethical guidelines were strictly followed, and patient samples were de-identified. Tumor samples were immediately washed 2–3 times with 5–10 ml of PBS/NSC basal medium to remove blood and debris, and the tissue was minced for 1–3 minutes with a No. 10 scalpel blade. The minced tissue was enzymatically dissociated by using 3–5 ml of pre-warmed Accutase® (Life Technologies) for 10–15 minutes in a 37 °C water bath. The solution was subsequently centrifuged, and 10–15 ml of basal medium was added to the tube and filtered through a 40 micron cell strainer to remove clumps and debris. After further washing cells were plated in NSC medium supplemented with 20 ng/ml EGF, 10 ng/ml bFGF and heparin (2 ng/ml), antibiotics added, and the cultures incubated at 37 °C in 5% CO_2_.

### Passaging and expansion of patient GBM derived neurospheres

The Methods used culture these neurosphere cell lines were published previously^43–46^. We cultured patient-derived neurosphere cell lines GBM4, GBM8, SK1035, SK987, SK892, SK429 and SK262 as previously described^43–46^. When the neurospheres reached an average size of 150–200 μm in diameter, subculture was initiated. The content of each flask was removed and placed in an appropriately sized sterile tissue culture tube, and centrifuged at 190 g for 6 min at room temperature. The supernatant was removed and the pellet dissociated to create a single cell suspension. The cell suspension was centrifuged, the supernatant was aspirated, and the cells resuspended in 1 ml of NSC medium and incubated at 37 °C in 5% CO_2_.

### Cell culture

Neurosphere lines were maintained on ultra-low attachment tissue culture plates (Corning) and grown in Neurocult Basal Medium (StemCell) supplemented with EGF (20 ng/mL), FGF (10 ng/mL), 0.002% Heparin, and 1% Pencillin/Streptomycin. Neurosphere lines were dissociated using Accutase digestion. 293 T and U251 cell lines were maintained in DMEM supplemented in 10% FBS, 1% Pencillin/Streptomycin. All cultures were confirmed to be mycoplasma free by PCR detection (Southern Biotech).

### Establishment of EC50 values

EC50 values were calculated according to procedures published previously^43–46^. GBM neurosphere cells were dissociated to single cell suspension and plated on ultra low-adherence 96 well plates at 2000 cells per well for drug testing. 12ADT and thapsigargin (Tg) drugs were serially diluted in log scale and added to 96 well plates to final concentration at 10, 1, 0.1, 0.01, 0.001, 0.0001, 0.00001 μM. After 72 hours of incubation with drug, the inhibition of cell growth was quantified by the Alamar Blue viability assay. Briefly, after incubation, Alamar Blue (#BUF012B, AbD Serotec) was added directly to the culture medium according to manufacturer specifications, and the fluorescence measured at 560/590 nm to determine the number of viable cells using TECAN (Infinite M200, Tecan Group Ltd.). Values were normalized to 0.00001 uM reading and EC50 values were determined by performing nonlinear regression analysis using commercially available software (Prism®, Graphpad Software, La Jolla, CA).

### CRISPR design

CRISPR plasmid design, generation, and validation were performed according to Methods and procedures published previously^11^. Briefly, for each gene of interest, two pairs of Cas9 guides were designed using the CHOPCHOP (Montague, TG Nucleic Acids Res 2014) software (available at http://chopchop.cbu.uib.no/) CRISPR guide sequences and primers against target region are detailed in Supplementary Table 2. Guides were cloned into the SpCas9–2A-GFP (px458) backbone modified to contain an EIF1α promoter (px458-ef1α)^47^. Briefly, Cas9 guides were purchased as oligonucleotides from IDT. These oligonucleotide guide pairs (with overhangs 5’CACCG on the forward strand and 5’AAAC, 3’C on the reverse strand) were phosphorylated, annealed and ligated into *BbsI*-digested px458 backbone. The ligated plasmid was then transformed into DH5α bacteria and grown on Carbenecillin plates overnight at 37 C. Single colonies were picked and cultured overnight and the plasmids isolated by mini or midi-prep (Invitrogen), and sequence validated. Transfection of CRISPR plasmids: 293XT and U251 cells were grown in DMEM with 10% FBS. 24 h prior to transfection, 8×10^4 cells/cm^2^ were seeded onto 6-well plates. The following day, the cells were transfected with the guide-containing px458-ef1a plasmids using Lipofectamine 3000 (ThermoFisher) according to manufacturer protocol. 72 h post-transfection, cells were FACS sorted for GFP + expression. Cells were then cultured in DMEM with 10% FBS with Pen/Strep for at least 1 week prior to validation and use in downstream analysis. To demonstrate Cas9 efficiency genomic DNA (gDNA) was isolated and PCR amplified using GoTaq (Promega) according to manufacturer instructions. To validate knockout of *ERN1*, western blot analysis was performed. Briefly, lysates of each cell line were prepared, separated by PAGE, transferred to a PVDF membrane, probed with monoclonal antibodies specific for IRE1α (Cell Signaling) or ACTB (Sigma), and imaged using HRP-conjugated secondary antibodies paired with Clarity Western ECL Substrate (BioRad). Since high quality monoclonal antibodies are not readily available for IGFBP3 or IGFBP5, we were unable to confirm knockout of these genes at the protein level. Instead, genomic DNA was isolated and PCR amplified with GoTaq (Promega) according to manufacturer instructions, using primers designed to flank the deleted region of each gene (Supplementary Table 2). PCR product was then resolved on a 2% agarose gel and imaged under UV.

### Flow cytometry

To determine cell viability, neurosphere cell lines or adherent cells lines were enzymatically dissociated through Accutase solution (Sigma) or TrypLE solution (Gibco), respectively. Cells were then washed in dPBS containing 1% BSA and 0.01% sodium azide and resuspended in wash buffer containing 7AAD at 20 ug/ml. Apoptosis assays were performed on single-cell suspensions, stained with fluorescein isothiocyanate (FITC) - conjugated Annexin V and propidium iodide (PI), following the FITC Annexin V Apoptosis Detection Kit (BD Biosciences). Surface staining for IGF1-R was performed using the commercially available conjugated antibody for CD221 (eBioscience). PSMA status was determined using a commercially available PE-conjugated antibody (BioLegend). All flow data were acquired on a FACSCalibur flow cytometer (BD Biosciences) and analyzed using CellQuest Pro (BD Biosciences) and FlowJo software (Tree Star). Procedures used follow procedures published previously^11^.

### Molecular biology

Cells were harvested, dissociated, and resuspended in dPBS. For RNA-seq preparation, total RNA was extracted using RNeasy Mini isolation kit (Qiagen). For RT-qPCR analysis, RNA was harvested using Nucleospin RNA (Macherey-Nagel). RNA quality and purity were determined using Nanodrop and normalized to equal concentrations to generated cDNA using High-Cap cDNA (Applied Biosystems). Endogenous controls of β-actin or the ribosomal subunit 18 s were used and relative quantification (RQ) expression was determined using the –ΔΔCT method. Methods are according to previously published protocols^11^.

### RNA-seq analysis

RNA was extracted from responder and non-responder cells using the Nucelospin RNA kit (Macherey Nagel). RNA sample purity was ascertained by the Nanodrop quantification method. Single end stranded RNA libraries were sequenced on an Illumina HiSeq. 4000. All samples and replicates were sequenced together on the same run. RNA-seq transcript quantification was performed with Sailfish version 0.9.1 using human reference transcriptome GRCh38 from Ensembl (URL: http://ftp.ensembl.org/pub/current_fasta/homo_sapiens/cdna/Homo_sapiens.GRCh38.cdna.all.fa.gz) and default parameters. We performed principal component analysis across all genes in SciKit-Learn version 0.19.1. Differential gene expression for 85 UPR genes from three Reactome pathways (R-HSA-381042.1, R-HSA-381038.2, R-HSA-381183.2) was determined using T-tests, implemented with Python package Scipy version 0.19.1 and p-values were adjusted for multiple testing using the Benjamini-Hochberg method. To obtain aggregate UPR expression values, unsigned z-scored expression values were summed across UPR genes in each replicate. WGCNA analysis was performed on whole transcriptome profiles with default parameters using the WGCNA R package version 1.61. Correlation of WGCNA modules with trait (Fig. 3A) was assessed based on the average Pearson correlation of module member gene expression level with sample classification using a 1 versus rest strategy such that correlation was first assessed for responders versus both non-responders and controls, then for non-responders versus both responders and controls and so on. To generate clustered heatmaps, expression values were clustered using agglomerative hierarchical clustering (Wards method) as implemented in the Seaborn clustermap function. All the Python analysis was done using Python version 2.6.

### Western blotting

Methods used were consistent with previously published protocols^11^. Briefly, cell lysates were harvested at specified time point and washed with ice-cold dPBS and suspended in RIPA lysis (Santa Cruz Biotechnology), supplemented with Halt Protease Inhibitor (Thermo). Cell lysates were lysed for 15 minutes on ice and centrifuged at 14,000 g for 15 min, and the supernatants were collected. Lysate protein concentration was determined using the Pierce BCA Protein Assay Kit (Thermo Scientific). Samples were heat-denatured, and equal concentrations of protein were loaded onto a 4 to 20% Mini-PROTEAN TGX Precast Gels (Bio-Rad), electrophoresed, and transferred PVDF membranes in tris-glycine transfer buffer containing 20% methanol. Transfer membranes were blocked with 5% nonfat milk in tris-buffered saline (TBS) containing 0.1% Tween 20 (TBS-T) for 1 hour at room temperature. The membranes were then incubated with the specified primary antibodies overnight at 4 °C. Membranes were subsequently washed three times for 5 min at room temperature with TBS-T and incubated with a HRP–labeled secondary antibody in 5% nonfat milk for 1 hour at room temperature. Membranes were then three times washed with TBS-T in five minute intervals. Bound antibodies were detected by chemilluminescence reaction using Pierce ECL Blotting Substrate (Thermo). The following primary antibodies were used: mouse monoclonal antibody to human GRP78 (BD Biosciences), rabbit monoclonal antibody to human PERK (Cell Signaling Technology), rabbit monoclonal antibody to phospho-eIF2α (Ser^51^) (Cell Signaling Technology), rabbit polyclonal antibodies to human ATF4 (CREB-2) (Santa Cruz Biotechnology), and HRP- conjugated goat antibodies to GAPDH (Santa Cruz Biotechnology). Secondary antibodies were HRP-conjugated anti-mouse IgG or anti-rabbit IgG (Santa Cruz Biotechnology).

### Bioinformatic analyses

(a) Projecting 12ADT response using TCGA GBM samples. In total six responder samples and 10 non-responder samples (treating replicates as independent samples) were used as a training set to fit a linear support vector machine classifier, using z-scored log2 TPM values for IGFBP3, IGFBP5 and ERN1 as features. Due to the small sample size, to evaluate generalization error we applied a leave one sample out (both replicates) strategy to evaluate this simple model. This model was then applied to predict potential to respond to 12ADT in 143 GBM samples in the TCGA. Prior to applying the model, GBM RNAseq data obtained from the Genomic Data Commons were processed to TPM using Sailfish version 0.9.1, log2 transformed and z-scored. The linear SVC model was implemented using the svm.LinearSVC module from the sklearn package version 0.19.1, python version 2.7.15. (b) Survival analysis. The following analysis was performed using. The regression analysis was performed using the survival package version 2.44–1.1 in R version 3.6.1. Progression-free survival (PFS) was calculated according to^48^. TCGA samples were divded into low, intermediate and high expression groups according to the 30^th^ and 70^th^ percentile of expression. Progression-free survival was then compared between the low and high expression groups using a one-tailed log-rank test, implemented using the logrank test function, specifying a one-tailed test using alternative = “less”, under the coin package, version 1.3–1.

## Supplementary information

Supplementary Information.

Supplementary Dataset.
