# Supplementary material for: IRE1α and IGF signaling predict resistance to an endoplasmic reticulum stress-inducing drug in glioblastoma cells
Source: Sci Rep. 2020 May 20;10:8348. doi: 10.1038/s41598-020-65320-6 (PMC7239929; doi:10.1038/s41598-020-65320-6)
Supplement: Supplementary file 1 — Supplementary Information. [file 41598_2020_65320_MOESM1_ESM.docx]

**Supplementary Information**

IRE1α and IGF signaling predict resistance to an endoplasmic reticulum stress-inducing drug in glioblastoma cells

By

Jeffrey J. Rodvold

Su Xian

Julia Nussbacher

Brian Tsui

T. Cameron Waller

Stephen C. Searles

Alyssa Lew

Pengfei Jiang

Ivan Babic

Natsuko Nomura

Jonathan H. Lin

Santosh Kesari

Hannah Carter

Maurizio Zanetti

Fig S1: **Responder GBM lines are not sensitive to G-202 and have low PSMA expression**. (A) Flow cytometric analysis of percent live (7AAD-) responder GBM8 neurosphere cells after 48 hour treatment with the pro-drug G-202 or its active form, 12ADT. (B) Expression of Prostate-specific membrane antigen (PSMA) in responder GBM4 and GBM8 lines as determined by flow cytometry. LNCaP cells used as positive control.


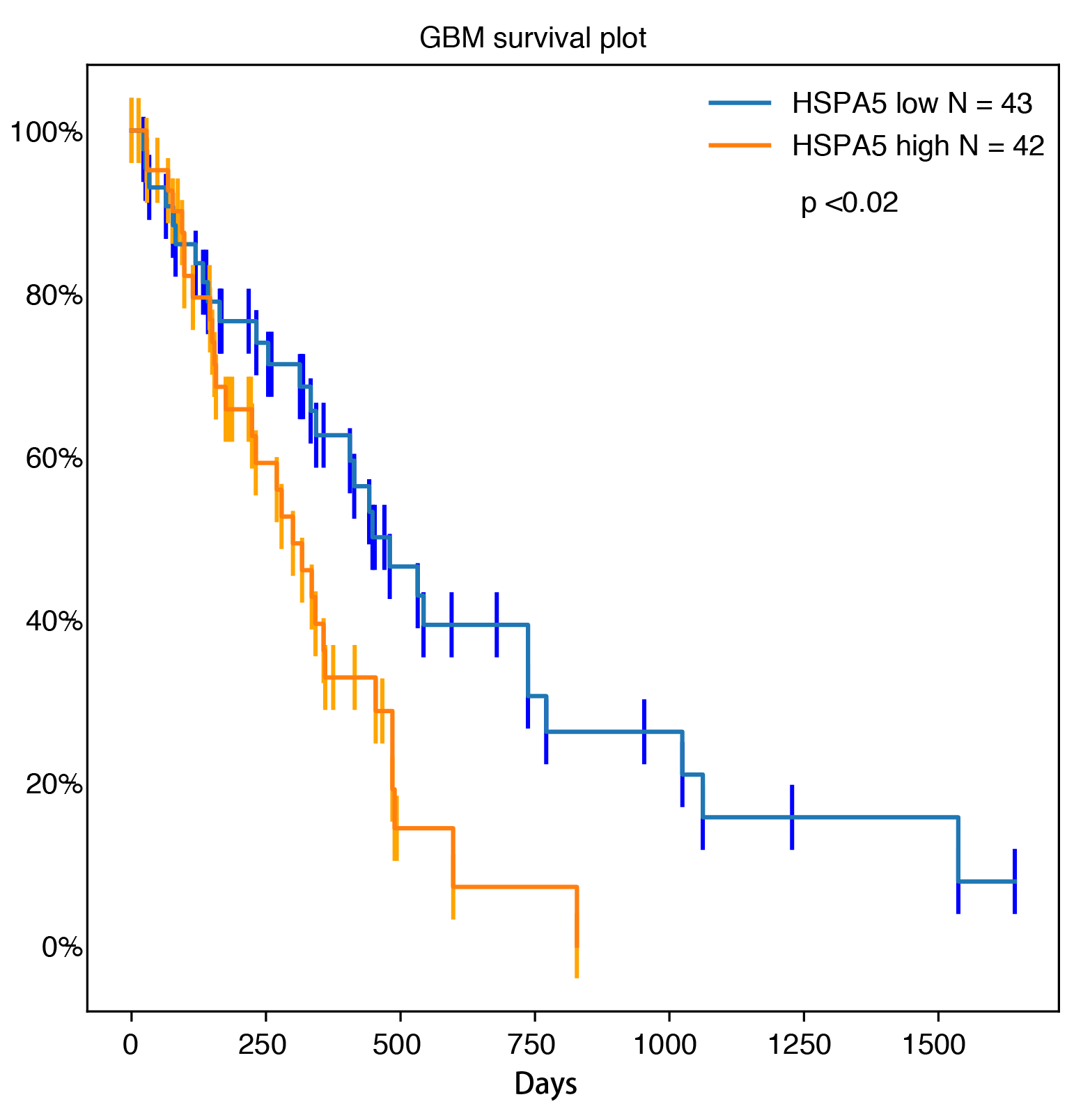


**Fig. S2**. **Survival analysis of GBM samples from TCGA.** Survival plot showing a significant difference (p<0.02) between HSPA5 high expression group (>70% quantile; n=42, orange) and HSPA5 low expression group (<30% quantile; n=43, blue) in GBM.


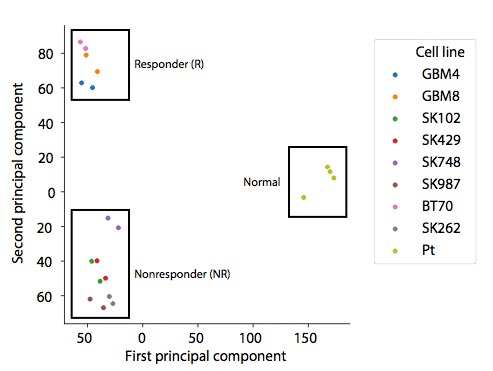


**Fig. S3.** **Transcriptome-wide principal component analysis distinguishes groups of GBM lines.** Scatter plot showing the first two components of the principal component analysis (PCA). Different cell lines with replicates are labeled with colors.

**Fig. S4.** **Differential expression patterns of MeBrown module genes detected by WGCNA.** Heatmap displaying differential expression patterns across genes within MeBrown module detected by WGCNA. Unsupervised clustering shows clear differences in expression patterns between responder (R) and nonresponder (NR) groups.

**Fig S5.** **Nonresponder GBM neurosphere lines are deficient in CDKN2A**.  (A) Differential expression of *CDKN2A* in responder and nonresponder groups. (B) Intracellular flow cytometric detection of expression of CDKN2A/p16 in permeabilized responder and nonresponder cells. HeLa cells are used as positive (+) control.


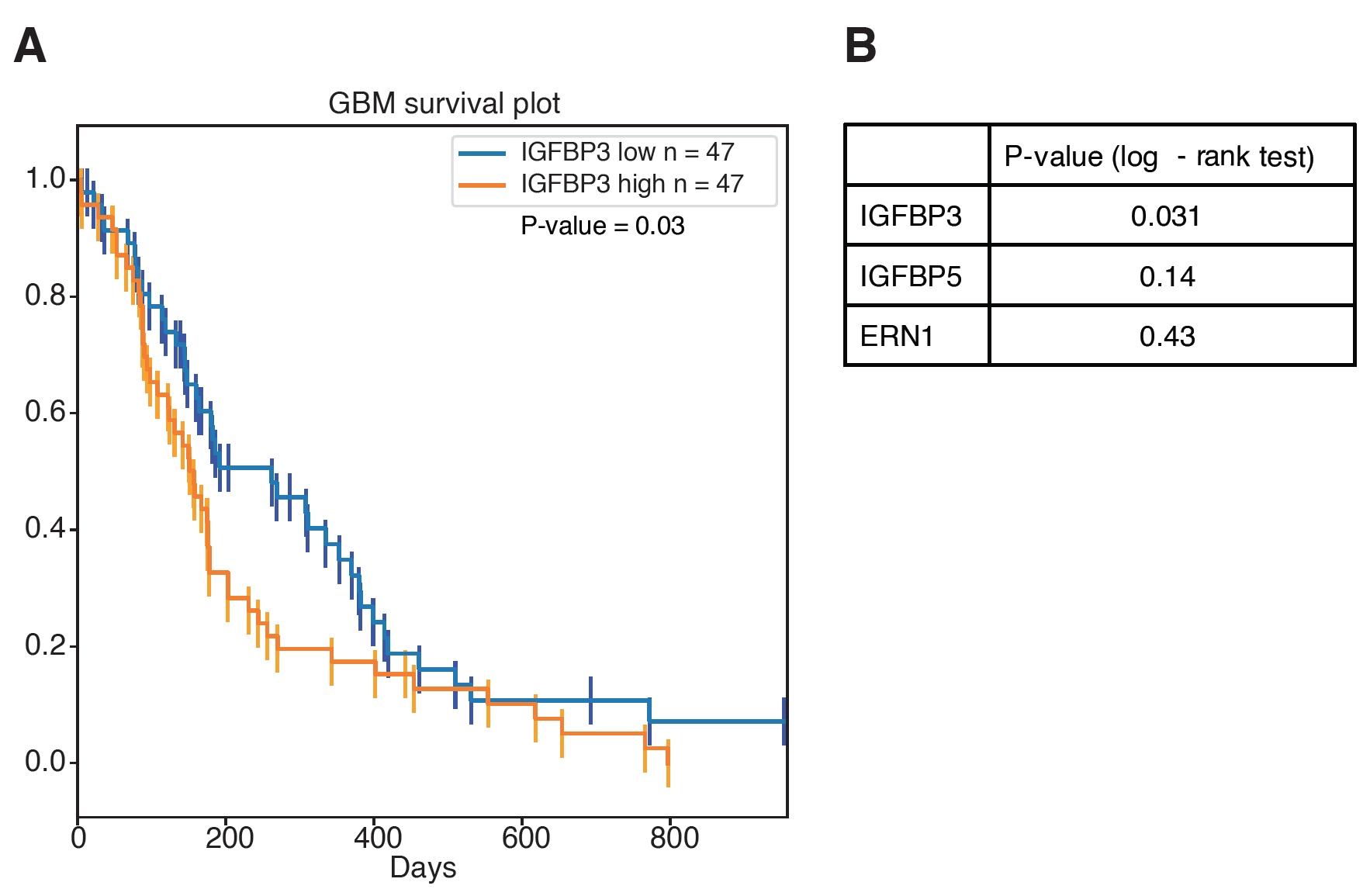


**Fig S6.** **Progression free survival (PFS) data of GBM samples (n = 94) from TCGA.**


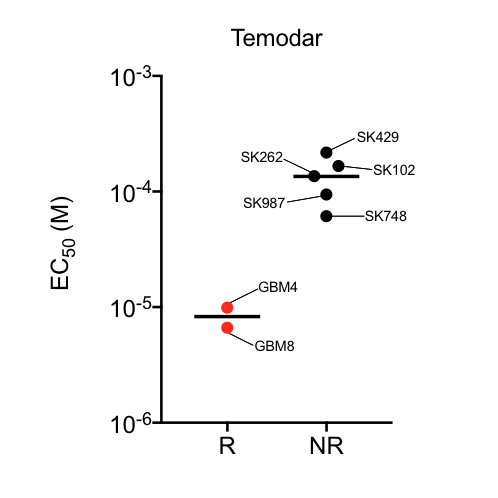


**Fig S7.** **GBM neurosphere lines are differentially sensitives to temozolomide (temodar).** Distribution of EC50 values between Responder (R) and Nonresponder (NR) neurosphere lines treated with Temodar for 72 hours and processed for viability by Alamar Blue absorbance. Each data point is a single experiment with three replicates, and is representative of two independent experiments.

**TABLE 1**

Excel document

**TABLE 2**

List of guides and primers used for CRISPR detection

|  | **ATF4** | **ERN1** | **IGFBP3** | **IGFBP5** |
| --- | --- | --- | --- | --- |
| Forward Guide | caccgGGATTTGAAGGAGTTCGACT | caccgAGGTGGGGCGCATCACAAAG | caccgTCGGAGGAAGACCGCAGCGC | caccgGACCCAGTCCAAGTTTGTCG |
| Reverse Guide | aaacAGTCGAACTCCTTCAAATCCc | aaacCTTTGTGATGCGCCCCACCTc | aaacGCGCTGCGGTCTTCCTCCGAc | aaacCGACAAACTTGGACTGGGTCc |
| fwd primer | tgggatctagggttaggggc | tttgtgtccaatggtgatggg | tgacctggttgcaacgttaag | agagagactcccgtgagca |
| rev primer | tttggagagcccctggtaga | caactcacgtcagcttgctct | cttgccctcctcctttaacaa | atgagggaatccccgagatg |
| predicted PCR size uncut | 574 | 259 | 350 | 253 |
| guide-guide size | 339 | 145 | 154 | 98 |
| predicted PCR size cut | 235 | 114 | 196 | 155 |
| expected shift | 339 | 145 | 154 | 98 |
