# Supplementary material for: IRE1α and IGF signaling predict resistance to an endoplasmic reticulum stress-inducing drug in glioblastoma cells
Source: Sci Rep. 2020 May 20;10:8348. doi: 10.1038/s41598-020-65320-6 (PMC7239929; doi:10.1038/s41598-020-65320-6)
Supplement: Supplementary file 2 — Supplementary Dataset. [file 41598_2020_65320_MOESM2_ESM.pdf]

Supplementary Data Set 1

IRE1α and IGF signaling predict resistance to an endoplasmic reticulum stress-inducing drug in glioblastoma cells

By

Jeffrey J. Rodvold
Su Xian
Julia Nussbacher
Brian Tsui
T. Cameron Waller
Stephen C. Searles
Alyssa Lew
Pengfei Jiang
Ivan Babic
Natsuko Nomura
Jonathan H. Lin
Santosh Kesari
Hannah Carter
Maurizio Zanetti

Table 1
UPR genes differential expression between Responder and Nonresponder neurospheres

Gene Name	BH adjusted	Significant	Test Statistic
SEC31A	4.40E-05	TRUE	-8.6884641
ZBTB17	0.00020426	TRUE	-7.1669657
PPP2R5B	0.00034982	TRUE	-6.5771929
SEC61A1	0.00053499	TRUE	-6.1198181
EXTL3	0.00053499	TRUE	-6.0196046
TLN1	0.00111668	TRUE	-5.335913
DNAJB9	0.00202282	TRUE	-4.9481583
NFYB	0.00279351	TRUE	-4.7198144
EDEM1	0.00429447	TRUE	-4.3987436
SHC1	0.00671375	TRUE	-4.1268871
ADD1	0.02768003	TRUE	-3.304984
ATF4	0.03254492	TRUE	-3.1931207
ERN1	0.04126652	TRUE	-3.0448415
ARFGAP1	0.04202993	TRUE	-2.9967062
EXOSC1	0.04202993	TRUE	-2.9715705
HSP90B1	0.04202993	TRUE	-2.9376931
TATDN2	0.04202993	TRUE	-2.9343157
CCL2	0.04245642	TRUE	-2.8738437
EIF2AK3	0.04327263	TRUE	-2.8349744
DDIT3	0.05847803	FALSE	-2.6257439
SULT1A3	0.0957187	FALSE	-2.3500546
SEC61A2	0.0957187	FALSE	-2.3268657
SEC61B	0.0957187	FALSE	-2.3188837
SEC63	0.15240604	FALSE	-2.0416679
GFPT1	0.16467567	FALSE	-1.9748972
GSK3A	0.16467567	FALSE	-1.9494477
SEC61G	0.16467567	FALSE	-1.9430733
TPP1	0.16467567	FALSE	-1.9337646
SYVN1	0.17571222	FALSE	-1.8559833
ASNS	0.17571222	FALSE	-1.8551286
ACADVL	0.18012681	FALSE	-1.8211083
HDGF	0.18012681	FALSE	-1.7952813
LMNA	0.18012681	FALSE	-1.7909633
HERPUD1	0.18012681	FALSE	-1.7758971
GOSR2	0.18846693	FALSE	-1.7376402
DDX11	0.2061292	FALSE	-1.6615274
FKBP14	0.21896474	FALSE	-1.607534
DCTN1	0.28206995	FALSE	-1.4374634
HYOU1	0.3205653	FALSE	-1.342779
HSPA5	0.3205653	FALSE	-1.3335185
IGFBP1	0.34773491	FALSE	-1.2570791
CTDSP2	0.34773491	FALSE	-1.2460724

CXXC1	0.35336434	FALSE	-1.2240905
SSR1	0.35807038	FALSE	-1.2040238
YIF1A	0.4104057	FALSE	-1.10045
CXCL8	0.43150876	FALSE	-1.0541434
DNAJB11	0.4360436	FALSE	-1.0353585
PLA2G4B	0.50820705	FALSE	-0.9036155
NFYA	0.51406019	FALSE	-0.8696578
WFS1	0.55217114	FALSE	-0.8032778
WIPI1	0.55866379	FALSE	-0.7825421
ASNA1	0.58473953	FALSE	-0.7346639
MYDGF	0.63168194	FALSE	-0.659609
SEC62	0.65761417	FALSE	-0.5940409
KDELR3	0.65761417	FALSE	-0.589721
XBP1	0.66859729	FALSE	-0.5617433
SERP1	0.66859729	FALSE	-0.5444628
CALR	0.69393871	FALSE	-0.4944933
CUL7	0.74168782	FALSE	-0.4081554
ATF6	0.93748355	FALSE	-0.1081324
ATP6V0D1	0.94490188	FALSE	-0.0845896
EXOSC6	0.97523369	FALSE	0.03160439
SRPRB	0.84690677	FALSE	0.23578227
MBTPS2	0.75728808	FALSE	0.36351466
PDIA5	0.75728808	FALSE	0.36678229
MBTPS1	0.72884821	FALSE	0.43693079
DCP2	0.66859729	FALSE	0.53981818
ATF3	0.65761417	FALSE	0.5910654
EXOSC7	0.50820705	FALSE	0.88975935
EXOSC3	0.50820705	FALSE	0.89743264
DNAJC3	0.33451763	FALSE	1.29445544
TSPYL2	0.21896474	FALSE	1.60241778
KLHDC3	0.20055505	FALSE	1.68952696
EXOSC8	0.18012681	FALSE	1.78439385
PREB	0.17550922	FALSE	1.88381977
EXOSC4	0.15240604	FALSE	2.03749536
NFYC	0.04849617	TRUE	2.73935914
PDIA6	0.04328488	TRUE	2.81567696
KHSRP	0.04245642	TRUE	2.86451391
EIF2S1	0.04245642	TRUE	2.8943529
PARN	0.02768003	TRUE	3.33072387
EXOSC9	0.01584015	TRUE	3.65306393
EXOSC5	0.00346973	TRUE	4.55542662
DIS3	0.00091154	TRUE	5.52309075
EXOSC2	0.00069235	TRUE	5.76578573
